# Sleep features and central sensitization symptoms in primary headache patients

**DOI:** 10.1186/1129-2377-15-64

**Published:** 2014-09-26

**Authors:** Marina de Tommaso, Marianna Delussi, Eleonora Vecchio, Vittorio Sciruicchio, Sara Invitto, Paolo Livrea

**Affiliations:** 1 Neuroscience and Sensory System Department, Neurophysiopathology of Pain Unit, Basical Medical Sciences, Bari University, Policlinico General Hospital, Giovanni XXIII Building, Via Amendola 207 A, 70124 Bari, Italy; 2 DISTEBA Department, Salento University, Lecce, Italy

**Keywords:** Sleep, Primary headaches, Central sensitization, Fibromyalgia

## Abstract

**Background:**

Association between sleep disorders and headache is largely known. The aim of the present study was to evaluate sleep quality and quantity in a large cohort of primary headache patients, in order to correlate these scores with symptoms of central sensitization as allodynia, pericranial tenderness and comorbidity with diffuse muscle-skeletal pain.

**Methods:**

One thousand six hundreds and seventy primary headache out patients were submitted to the Medical Outcomes Study (MOS) within a clinical assessment, consisting of evaluation of frequency of headache, pericranial tenderness, allodynia and coexistence of fibromyalgia syndrome (FM).

**Results:**

Ten groups of primary headache patients were individuated, including patients with episodic and chronic migraine and tension type headache, mixed forms, cluster headache and other trigeminal autonomic cephalalgias. Duration but not sleep disturbances score was correlated with symptoms of central sensitization as allodynia and pericranial tenderness in primary headache patients. The association among allodynia, pericranial tenderness and short sleep characterized chronic migraine more than any other primary headache form. Patients presenting with FM comorbidity suffered from sleep disturbances in addition to reduction of sleep duration.

**Conclusion:**

Self reported duration of sleep seems a useful index to be correlated with allodynia, pericranial tenderness and chronic headache as a therapeutic target to be assessed in forthcoming studies aiming to prevent central sensitization symptoms development.

## Background

A strong relationship between insomnia and painful disorders has been reported [1], and studies indicate that pain not only might be a risk factor for insomnia but that the two disorders reciprocal influence and exacerbate each other [2]. It is also likely that when insomnia and chronic pain occur together their consequences are even more devastating [2]. In clinical studies acute painful stimuli applied to healthy subjects during sleep resulted in transient arousals [3], while chronic pain patients had poorer sleep than controls in terms of sleep latency, sleep efficiency and awakenings after sleep onset [4,5]. The existence of a correlation and/or comorbidity between sleep disorders and headache has been also largely demonstrated [6,7].

A recent review stated that sleep loss and sleep deprivation have severe effects on human health, being a risk factor for neurologic diseases, including headache [8]. Migraine attacks may be precipitated by sleep deprivation or excessive sleep and sleep is also associated with relief of migraine attacks. In previous studies, migraine attack was found to be precipitated by sleep deprivation in 24% and by sleep excess in 6% of cases [9]. The “lack of sleep” is endorsed as a trigger among 48% to 74% of migraineurs and 26% to 72% of tension-type headache sufferers, and sleep disturbance has been consistently identified as a headache trigger in retrospective studies [10,11]. The effect of sleep in terminating an attack of headache is also well known [7]. Kelman and Rains [6] assessed relations between sleep and migraine and found that approximately half of patients reported at least occasional symptoms of insomnia, 38% reported sleeping less than 6 hours per night, and 50% of patients reported that sleep disturbance triggered their migraines . The severity and prevalence of sleep problems increase proportionally to headache frequency, such that the majority of chronic migraine patients (68-84%) suffer from insomnia on a near-daily basis [12-14]. Central sensitization is a phenomenon of pain processing, which may predispose to chronic pain. Allodynia occurring during migraine attack and persistent pericranial tenderness in migraine and tension type headache are symptoms of central sensitization [15,16], which may be aggravated by sleep disturbances and/or exert a negative impact on sleep in a mutual negative implication. The comorbidity between primary headaches and fibromyalgia is also present in patients with accentuated tendency to central sensitization [17-19]. Sleep deprivation causes hyperalgesia in healthy volunteers [20], so the relationship between sleep disturbances and symptoms of central sensitization should be addressed in primary headache in order to optimize their management.

The aim of the present study was to evaluate sleep problems and duration in a large cohort of primary headache patients, in order to correlate these parameters with symptoms of central sensitization as allodynia, pericranial tenderness and comorbidity with diffuse muscle-skeletal pain. For this purpose the Medical Outcomes Study Sleep Scale [21] was employed, which is a generic measure of sleep problems that can be used to compare different clinical populations to one another on a common metric. The questionnaire is brief, responsive to change, and has been used in FM [22]. In this study we didn’t evaluate a control population, because our main interest was to correlate sleep scores with clinical symptoms of central sensitization in a large cohort of headache patients.

## Methods

One thousand six hundreds and seventy out patients were included in the present evaluation, among 2135 coming consecutively to the Neurophysiopathology of Pain center of Bari University since 1/2012 to 12/2013. Patients were included after three months from their first approach to our Department, when a visit date was assigned. Patients were invited to sign up their headache and the possible presence of vegetative symptoms and of allodynia, using a scale reporting the present of nausea, vomiting, phonophobia and photophobia, and the questionnaire reported by Ashkenazi et al. [23,24] and previously applied in Italian version [17,18].

The diagnosis was made by 2 neurologists expert in headache, in accord with the International Headache Society criteria, on the basis of headache characteristics and frequency in the three months preceding the visit [25,26]. During the first visit, patients underwent the clinical assessment, defined in previous studies [17,18], consisting of evaluation of diaries reporting frequency of headache, as the average number of days with headache/month, computed in the last three months, completed by the report of pain features and vegetative symptoms during single headache episodes. The allodynia questionnaire was also evaluated, which patients had been requested to use for each headache episode [23,24]. The presence of allodynia symptoms during the headache episodes was inferred from the notes the patients made on their diaries while answering the Italian version of the questionnaire we had given them to complete after each headache episode [24]. The most recent IHS criteria [26] were directly applied to cases come in the last study year, while previous diagnoses were updated by means of a retrospective evaluation of headache characteristics and frequency.

The inclusion criterion was the diagnosis of a primary headache syndrome, according to the IHS criteria [25,26]. Patients with general medical and/or other neurological or psychiatric diseases were excluded from the study, as well as patients on central nervous system-active drug or preventive treatment for primary headache. Patients with a diagnosis of “probable” primary headache were excluded. We considered groups including at least 10 patients. For cluster headache patients [22,23], only patients with chronic form or during cluster episode were included.

Patients were classified as allodynic if they confirmed the presence of at least one symptom reported in the questionnaire, for 50% or more of the headache episodes. For each patient a mean allodynia score was also computed. Total tenderness score (TTS) was also evaluated in all patients to evaluate pericranial tenderness, following the procedure described by Langermark and Olesen [27]. Assessment was performed with manual palpation by a neurologist with experience in headache, who was experimentally blinded to the patient’s diagnosis. The right frontal muscle, masseter muscle, temporal muscle, pterigoid muscle, sternocleidomastoid muscle, sternocleidomastoid muscle insertion, neck muscle insertion and trapezium muscle were examined using the TTS system. Patients were submitted to the depression [self-rating depression scale (SDS)] and anxiety. [Self-rating anxiety scale (SAS)] scales, as they are considered reliable tools to detect symptoms of anxiety and depression in a general non-psychiatric patient population [28,29]. According to previous studies [17] we applied the Italian version of the MIDAS score to all type of headaches [30,31], to quantify headache-related disability.

In order to assess the presence of fibromyalgia comorbidity, patients underwent the most recent diagnostic criteria [32], together with fibromyalgia impact profile (FIQ) [33] and tender point count [34]. The Fibromyalgia Impact Questionnaire (FIQ) is a fibromyalgia-specific patient-reported outcome Instrument designed to assess health status, progress and outcomes in patients with fibromyalgia. It contains 10 subscales that are combined to yield a total score [33]. The Manual Tender Point Survey (MTPS) provided a rate of the severity of pain patients felt upon palpation of the specific 18 tender points defined by the American College of Rheumatology and of three control sites [34]. In all patients the Medical Outcomes Study (MOS) [21] was applied, which is a 12-item self report questionnaire that measures six dimensions of sleep, including initiation, maintenance (e.g. staying asleep), quantity, adequacy, somnolence (e.g. drowsiness), and respiratory impairments (e.g. shortness of breath, snoring, in a total of eight parameters. It has been previously applied in patients with chronic pain [19] and primary headache [14,15]. Each scale (except sleep quantity) is recalibrated onto a 0–100 scale. For most scales, higher scores indicate worse sleep problems. For sleep quantity (SLPQRAW) lower scores indicate worse sleep problems, referring to hours of sleep for night in the last week. The MOS Sleep Scale can be aggregated to produce 2 summary indices, the Sleep Problems Index II (9 items) and the Sleep Problems Index I (six items). Each of these indices integrates the domains of sleep disturbance, sleep adequacy, shortness of breath, and somnolence into a single score. The difference between Sleep Problems Index 1 and 2 is simply length rather than domain coverage; potentially overlapping items were eliminated in Index 1, which seems a reliable and simple global sleep problems score. Higher scores on either index are indicative of worse sleep problems. In this study we choose to report results obtained by sleep quantity (SLPQRAW) and Sleep Problems Index 1 (SLP6), while the results of the other items were reported in the Additional file 1.

The study was approved by the Bari Policlinico General Hospital ethical committee, and each patient signed an informed consent.

### Statistical analysis

The multivariate ANOVA was applied considering the parameters SLPQRAW (sleep quantity -raw) and SLP6 (sleep problems index I), allodynia and pericranial tenderness as variables and the type of headache as factors. The Bonferroni was employed as post-hoc test, to compare the MOS SLPQRAW and SLP6 items, allodynia and pericranial tenderness between the single headache groups. Results obtained by the comparison of the other MOS items were reported in the Additional file 1. The presence of allodynia in headache groups was further compared by means of chi-square test, and the SLPQRAW and SLP6 items compared between allodynic and not allodynic patients by means of multivariate ANOVA. Patient was considered allodynic when reporting at least one symptom in more than 50% of headache episodes.

The Pearson correlation test was also employed to correlate the MOS items with allodynia and pericranial tenderness in the total of patients and single primary headache groups including at least 100 cases. In this correlation, we included also the anxiety and depression scores. For the high number of correlations, we considered only Pearson values with a level of significance <0.01. Correlation analysis regarding the other MOS items was reported in Additional file 1.

A multivariate ANOVA was also employed considering the two MOS SLPQRAW and SLP6 parameters as variables and the presence of fibromyalgia diagnosis as factor. The Pearson correlation test was also employed to test correlations among MOS items and fibromyalgia impact questionnaire and pain at tender points. Statistic was computed by IBM SPSS vers 21. For MANOVA tests, age was introduced as correcting factor and sex as a covariate.

## Results

Ten groups of primary headache patients were individuated, which demographic and clinic characteristics are detailed in Table 1. The primary forms with fewer than 10 cases were included in a single group as “other primary headaches”, which was quite heterogeneous (Table 1).

**Table 1:** Demographic and clinical features of primary headache patients

	**Sex**	**Age**	**Duration (years) M ± SD**	**Headache frequency (days with headache/month) M ± SD**	**MIDAS M ± SD**	**TOTAL TENDERNESS SCORE M ± SD**	**ALLODYNIA M ± SD**	**SLP6 M ± SD**	**SLPQRAW M ± SD**
Migraine without aura 1.1	175 M	37.09	16.23	6.45	30.16	4.97	3.18	49.14	6.67
	625 F	12.21	11.75	5.32	36.61	5.74	2.16	24.91	1.5
Migraine with aura 1.2.1	11 M	35.87	12.97	2.32	13	3.47	2.38	43.44	7.07
	27 F	12.15	10.15	2.68	21.33	4.98	2.62	29.09	1.17
Migraine Without aura 1.1 plusEpisodic tension type headache 2.2	24 M	38.56	16.28	6.2	25.16	4.92	3.36	53.54	6.83
	78 F	13.21	11.41	5.8	32.14	4.8	2.27	24.30	1.83
Migraine without aura 1.1. plus migraine with aura 1.2.1	10 M	35.98	17.15	5,34	28	5.64	3.36	50.58	7.04
	57 F	10.67	10.2	5	27.19	5	2.35	22.35	1.53
Chronic Migraine 1.3	53 M	41.34	18.9	23.52	61.47	7.84	3.62	52.46	6.1
	280 F	14.4	13.7	6.5	47.3	6	2.34	21.67	1.63* + ^°
Episodic tension type headache 2.2	35 M	40.64	12.28	6.12	16.50	4.66	2.55	52.1	6.86
	76 F	16.64	12.46	4.12	25.11	4.37	2	22.3	1.76
Chronic tension type headache 2.3	44 M	43	12.77	24,19	45.13	6	2.48	50.28	6.31
	89 F	14.98	13.33	6.45	53.03	5.66	2.28	23.33	1.67
Other TACs (Paroxysmal hemicranias 3.2, n° 8; Hemicrania continua, 3.4 n° 9)	4 M	43.29	13.73	26.22	38.47	3.67	3.71	43.80	6.73
	13 F	16.13	14.42	9.3	39.25	3.81	2.73	25.58	2.27
Cluster headache 3.1 (3.1.1 episodic cluster headache, n° 18; 3.1.2 chronic cluster headache, n° 10)	21 M	39.21	14	11.6	27	2.43	2.74	52.5	5.95
	7 F	11.52	11.5	14.5	35.34	3.02	2.45	23,48	2.05
Other primary headaches (Primary exercise headache 4.2, n° 5; Primary headache associated with sexual activity 4.3, n° 3; Primary thunderclap headache 4.4, n° 4; Primary stabbing headache 4.7, n° 9; Nummular headache 4.8, n° 7; Hypnic headache 4.9, n° 5)	14 M	41.71	11.86	14.11	22.9	2.96	1.4	54.37	6.45
	27 F	15.94	12.7	12.13	39.9	3.2	1.6	24.47	1.27

SLPQRAW (Sleep quantity -raw); SLP6: Sleep problems index I.

The IHS classification code is specified for each headache group and type. The results of Bonferroni test are shown: Chronic migraine vs * migraine without aura, + migraine without aura plus episodic tension type headache, ^migraine without + plus migraine with aura ° episodic tension type headache: p < 0.05.

**Figure 1:**
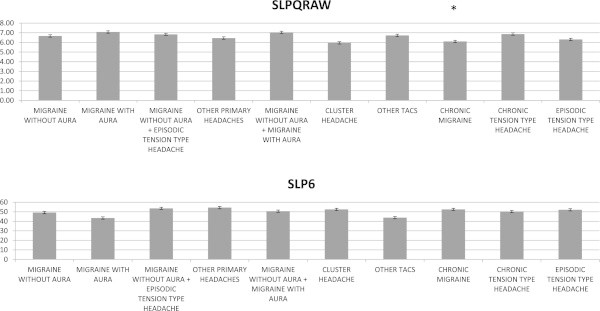
**Mean values and standard errors of SLPQRAW (sleep quantity -raw) and SLP6 (sleep problems index 1) in primary headache groups.** The symbol * indicate the results of Bonferroni test: chronic migraine compared to migraine with and without aura, other TACs, migraine without aura plus episodic tension type headache, and chronic and episodic tension type headache: p < 0.05.

The MANOVA analysis indicated that the SLPQRAW (sleep quantity -raw) was significantly different (F = 5.87 DF 9 p < 0.0001), while the SLP6 index was similar.

(F = 0.93 DF 9 n.s.), among headache groups. The Bonferroni test showed that chronic migraine reported fewer sleep time compared to episodic migraine without aura, episodic tension type headache and the groups associating migraine without aura plus migraine with aura and tension type headache (Table 1, Figure 1). The other MOS scores were not significantly different among primary headache groups (see Additional file 1). Sex did not influence sleep scores (SLPQRAW F = 3.28 DF 1 n.s.; SLP6 F = 1.55 DF 2 n.s.).

**Figure 2:**
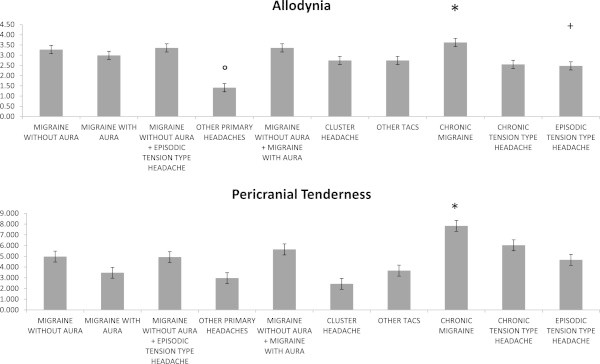
**Top: Mean values and standard errors of allodynia scores in primary headache groups.** The symbols *, ° and + indicate the results of Bonferroni test: * chronic migraine compared to migraine with and without aura, other TACs, migraine without aura plus episodic tension type headache, chronic and episodic tension type headache: * p < 0.05; ° other primary headaches compared to all the remaining groups: p < 0.05; + episodic tension type headache vs migraine without aura: p < 0.05. Bottom: Mean values and standard errors of pericranial tenderness scores in primary headache groups. The symbol * indicates the results of Bonferroni test: * chronic migraine compared to other primary headache groups, excluding chronic tension type headache: p < 0.05.

Allodynia scores were significantly different among headache groups (F 5.13 DF 9 p <0.0001), for lower values in other primary headaches compared to other groups, excluding tension type headache, and significant higher scores in chronic migraine compared to chronic and episodic tension type headache. Episodic tension type headache displayed reduced scores of allodynia also in comparison with migraine without aura (Table 1, Figure 2).

**Figure 3:**
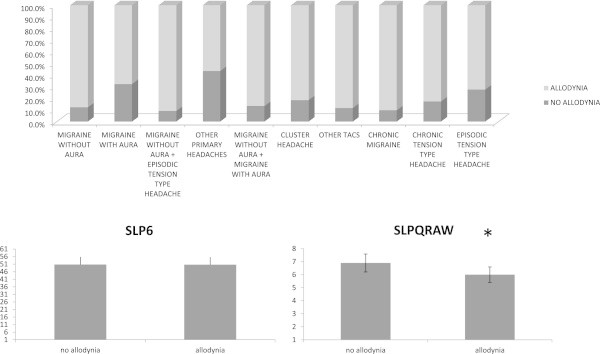
**Top: Percent rates of primary headache patients presenting with allodynia.** Bottom: Mean ± SE SLPQRAW (sleep quantity -raw) and SLP6 (sleep problems index 1) scores in primary headache patients presenting and not presenting with allodynia. Results of MANOVA are shown: * p < 0.05.

Pericranial tenderness scores were also significantly different among headache groups (F = 8.82 DF 9 p < 0.0001), being higher in chronic migraine compared to other headaches, excluding chronic tension type headache. (Table 1, Figure 2). Sex significantly influenced both allodynia and TTS scores, for higher values in females (allodynia F = 13.49 DF 1 p < 0.0001; TTS F = 7.97 p 0.005).The presence of allodynia varied from the 56.4% in the group of other primary headaches to the 91% in chronic migraine (chi square: 66.94 DF 9 p < 0.0001). The SLP6 item was similar in allodynic and not allodynic patients (F = 1.94 n.s.), while the SLPQRAW score was significantly lower in allodynic patients (F = 4.5 p 0.039) (Figure 3).

**Figure 4:**
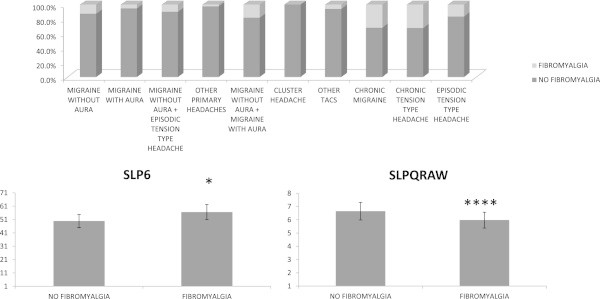
**Top: Percent rates of primary headache patients presenting with Fibromyalgia syndrome.** Bottom: Mean ± SE of SLPQRAW (sleep quantity -raw) and SLP6 (sleep problems index 1) scores in primary headache patients presenting and not presenting with allodynia. Results of MANOVA are shown: *p < 0.05, ****p < 0.00001.

The SLPQRAW was significantly correlated with all clinical features, including disease duration, allodynia, TTS, SAS and SDS (Table 2), while the SPL6 index was not correlated to headache frequency or symptoms of central sensitization, showing a significant correlation with anxiety. Allodynia and pericranial tenderness were also significantly correlated with anxiety and depression scores, as well as with invalidity linked with headache in the total and single headache groups (Table 3). The same correlations were also present in chronic migraine group, while in episodic migraine without aura and chronic tension type, the total time of sleep was inversed correlated with pericranial tenderness (Table 3). The other MOS scores were not correlated with allodynia and pericranial tenderness (See Additional file 1).Fibromyalgia was diagnosed in the 17, 54% of primary headache patients. The percent number of FM patients for single headache groups is reported in Figure 4. The frequency of FM ranged from 32, 33% in chronic tension type headache, to 0% in cluster headache (chi square 104.12 DF 9 p 0.00001).The MANOVA showed that the SLPQRAW MOS score was significantly different between patients with and without FM symptoms (39.15 DF 1 p <0.0001). Sleep problems, expressed by the SLP6 index, were also slightly increased in patients presenting with FM comorbidity (F 4.1 DF 1 p 0.047) (Figure 4).

**Table 2:** Pearson correlation analysis among MOS items and main clinical features in the total of primary headache patients and main subgroups

**SLPQRAW**	**SLPQRAW**	**SLP6**	**DURATION**	**FREQUENCY**	**MIDAS**	**ALLODYNIA**	**TTS**	**SAS**	**SDS**
All (n°1670)	1	0.019	-0.180**	-0.144**	-0.141^**^	-0.085^**^	-0.117^**^	-0.208^**^	-0.233^**^
Migraine (1.1) (n° 800)	1	0.001	-0.164**	-0.079*	-0.083*	-0.043^*^	-0.185**	-0.185^**^	-0.213^**^
Migraine (1.2 and 1.2 plus1.1) (n°105)	1	0.002	-0-060	-0.121	**-**0.111	-0.110	-0.210*	-0.112	-0.154
Chronic Migraine 1.3 (n°333)	1	0.046	-0.136*	-0.274**	-0.287**	-0.332**	-0.321**	-0.312**	-0.356**
Chronic tension type headache 2.3 (n°133)	1	0.034	-0,193	-0.189	-0.234*	-0.122	-0.238**	-0.125	-0.259**
Episodic tension type headache 2.2 (n°111)	1	0.023	-0,211*	-0.123	0.145	0.167	0.145	0.176	-0.258**
SLP6									
All (n°1670)	0.019	1	0.09	0.047	0.028	0.010	0.055	0.087^**^	0.060
Migraine (1.1) (n° 800)	0.001	1	0.087	0.029	0.021	0.008	0.045	0.099	0.097
Migraine (1.2 and 1.2 plus1.1) (n°105)	0.004	1	0.121	0.023	0.012	0.005	0.034	0.134	0.135
Chronic Migraine 1.3 (n°333)	0.023	1	0.097	0.123	0.121	0.034	0.045	0.145*	0.090
Chronic tension type headache2.3 (n°133)	0.189	1	0.081	0.034	0.089	0.076	0.029	0.089	0.129
Episodic tension type headache 2.3 (n°111)	0.056	1	0.101	0.89	0.034	0.109	0.115	0.178	0.276

The IHS specification code is indicated: 1.1 migraine without aura; 1.2 migraine with aura; 1.3 chronic migraine; 2.3 chronic tension type headache; 2.2 episodic tension type headache.

The significant results are outlined in bold character. **p < 0.01. The p values < 0.05 are indicated by the symbol *, but not outlined.

SLPQRAW: Sleep quantity raw score.

SLP6: Sleep index 1.

TTS Total Tenderness Score.

SAS: Self rating Anxiety Scale.

SDS: Self rating Depression Scale.

**Table 3:** Pearson correlation analysis among central sensitization symptoms and other clinical features in the total of primary headache patients and main subgroups

**ALLODYNIA**	**FREQUENCY**	**MIDAS**	**ALLODYNIA**	**TTS**	**SAS**	**SDS**
All (n°1670)	0.085^*^	0.296^**^	1	0.228^**^	0.296^**^	0.292^**^
Migraine (1.1) (n° 800)	0,103**	0.213^**^	1	0.133^**^	0.274^**^	0.207^**^
Migraine (1.2 and 1.2 plus1.1) (n°105)	0.204*	0.223*	1	0.225^*^	0.284^**^	0.374^**^
Chronic Migraine 1.3 (n°333)	0.321**	0.345**	1	0.367**	0.267**	0.336**
Chronic tension type headache2.3 (n°133)	**0.09**	0.187*	1	0.289**	0.334**	0.310**
Episodic tension type headache 2.2 (n°111)	0,118	0,400**	1	0.280**	0.334**	0.362**
TTS						
All (n°1670)	0.199^**^	0.188^**^	0.228^**^	1	0.251^**^	0.216^**^
Migraine (1.1) (n° 800)	0.219^**^	0.213^**^	0.133^**^	1	0.203^**^	0.232**
Migraine (1.2 and 1.2 plus1.1) (n°105)	0.242*	0.326^**^	0.225*	1	0.292^*^°	0.327^**^
Chronic Migraine 1.3 (n°333)	0.180**	0.226^**^	0.367**	1	0.270^*^°	0.227^**^
Chronic tension type headache2.3 (n°133)	0.180*	0.226^**^	0.289**	1	0.269^*^°	0.228^**^
Episodic tension type headache 2.2 (n°111)	0,253*	0.133	0.280**	1	0.245*	0.362**

The IHS specification code is indicated: 1.1 migraine without aura; 1.2 migraine with aura; 1.3 chronic migraine; 2.3 chronic tension type headache; 2.2 episodic tension type headache.

The significant results are outlined in bold. **p < 0.01. The p values < 0.05 are indicated by the symbol *, but not outlined.

TTS Total Tenderness Score.

SAS: Self rating Anxiety Scale.

SDS: Self rating Depression Scale.

Fibromyalgia severity, measured by FIQ score [22] was also correlated with short sleep time and increased sleep problems (Pearson correlation: FIQ vs SLPQRAW -0.332 p < 0.01; vs SLP6 0.282 p < 0.01). Pain at tender point was not correlated with MOS items.

## Discussion

Our study tested sleep features and central sensitization symptoms in a large cohort of primary headache patients including patients with TACs and other mixed forms. The results of this study showed that sleep quantity was significantly reduced in primary headache patients reporting symptoms of central sensitization, as allodynia during headache episodes and pericranial tenderness. Chronic migraine was the headache group reporting more severe allodynia in respect to other headache groups, in accord with first descriptions of allodynia development during migraine attack [16] as a predictor of increasing frequency of migraine days [35]. However, allodynia was largely represented in chronic tension type headache and cluster headache groups, while a smaller part of the mixed primary headache group reported those symptoms. Current theories attribute to central sensitization an important role in tension type headache pathogenesis [36-38] with evidence of allodynia symptoms in chronic forms [38], as confirmed by present results. In regard to cluster headache, previous studies reported allodynia during cluster episodes, in accord with present results [39], while this is the first study suggesting the presence of allodynia in other TACs, as paroxysmal migraine and hemicranias continua. Actually we cannot advance conclusions on reduced expression of allodynia in the mixed headaches group, which was a very heterogeneous group and worthy of further case series enlargement, considering the importance of a better knowledge of these rare disorders. Unfortunately, cases of hyping headache were also few, deserving further consideration in a larger group. Pericranial tenderness was also more expressed in chronic migraine compared to other headache groups, excluding chronic tension type headache. This confirmed the relation between pericranial pain and chronic tension type headache and migraine [15] but suggested the scarce presence of this symptom in TACs and other primary headache forms. Quantity more than other sleep problems seemed to be critical for central sensitization symptoms severity in our headache series, with a strong correlation with both allodynia and pericranial tenderness. Gender was a critical factor for both allodynia and pericranial tenderness, but not for sleep features, according to complex hormonal influences on pain processing and chronic syndromes [40] Primary headaches may lead to sleep reduction in both males and females, with pronounced expression of central sensitization symptoms in the latter group. However, the influence of sex on primary headaches phenotype including symptoms of central sensitization is very complex and deserves specific examination in further studies. Chronic migraine patients presented with reduced sleep quantity compared to episodic migraine and tension type headache, confirming the strict relation between short sleep time, central sensitization symptoms and chronic pain development. The MOS questionnaire tests six dimensions of sleep, including initiation, maintenance (e.g. staying asleep), quantity, adequacy, somnolence (e.g. drowsiness), and respiratory impairments (e.g. shortness of breath, snoring). We choose to examine only the SLP6 index, which is a summary of sleep problems based on adequacy, shortness of breath, and somnolence [21]. Moreover, the other MOS scores testing sleep problems did not show relevant results from statistical comparison among headache groups and correlation with symptoms of central sensitization. In a study by Lovati et al. [41], headache patients presenting with respiratory problems during sleep, reported also high scores of allodynia. In light of present results, the increase of central sensitization symptoms could be related to the sleep deprivation induced by respiratory dysfunction. Angstrom et al. [42] found EEG signs of sleep deprivation in tension type headache and migraine patients, despite normal sleep times in diary. They concluded that headache patients need on average more sleep than healthy controls, and that inadequate rest might be an attack-precipitating- and hyperalgesia-inducing factor. In our headache patients, the sleep time was on average 6.7 hours in non allodynic and 6 hours in allodynic patients, which was below the limit of optimal sleep in accord with MOS score [43], actually speculative and worthy of confirmation in a control group. Sleep deprivation causes hyperalgesia in normal controls [20], while further case- control studies may clarify if the sleep time threshold for central sensitization symptoms development may be lower in primary headache in respect to controls. Moreover, the reasons for sleep time reduction are numerous in primary headaches, as showed by the correlation between short sleep time and headache frequency and severity, as well as anxiety and depression, though some significant correlations were due to the high number of subjects and not present in all primary headache groups. Sleep duration and headache frequency and invalidity were inversely correlated in chronic migraine patients, which included patients affected by more severe central sensitization symptoms and sleep deprivation. High frequency of headache may cause sleep disruption for unexpected night or early morning arousing, as well as for troubling in falling asleep, while “lack of sleep” is a trigger among the majority of migraineurs and tension-type headache sufferers [10,11]. Headache severity and invalidity, as tested by MIDAS, may further deteriorate sleep for the worries coming from social and familiar problems caused by high headache frequency. Psychopathological traits as anxiety and depression are well known factors of sleep reduction [44]. The SLPQRW score was also reduced in patients with long disease duration, probably because headache and especially migraine persistence may aggravate this aspect of sleep. In chronic migraine group, this correlation did not reach the statistic significance, as sleep deprivation may be intrinsic to this chronic syndrome just in the early phase. Headache severity, anxiety and depression were correlated with allodynia and pericranial tenderness in the total and single headache groups, also in accord to previous studies [45]. Moreover, chronic migraine and chronic tension type headache showed a relationship between short sleep duration and increased expression of central sensitization symptoms as pericranial tenderness, suggesting a reverberating mechanism where headache frequency, low pain threshold, reduced sleeps time and psychopathological factors interfere in a complex way in generating chronic syndromes [14]. In this sense, chronic migraine is caused by a critical convergence of these factors [12,14]. The management of headache could take into account therapeutic substances acting on pathophysiological mechanism of both sleep and pain [46] as well as behavioral non pharmacological approach to sleep modification [47].

A complex dysfunction in orexins transmission may also explain the correspondence between lack of sleep and altered modulation of pain with central sensitization facilitation. Chronic pain lead to sleep disturbance in parallel with changes in circadian rhythm for mRNA expression of orexin receptors in animal models of hypothalamus [48], while the analgesic role of orexins is currently recognized on the basis of the experimentally induced inhibition of nociceptive transmission via spinal orexin receptors activation [49].

The total score of sleep problems, as tested by the SLP6 index, did not show the same performance as the raw score of sleep duration in differentiating patients with signs of central sensitization. The reason may be a specific effect of sleep deprivation on mechanisms of central sensitization, as suggested by studies on healthy volunteers [20]. However, specific sleep disturbances may be present in primary headaches, as reported in previous studies, where patients with chronic headache showed a high prevalence of daytime sleepiness and snoring with respect to controls [14]. The SLPQWR score, with the limitation due to a subjective report, seems a useful index to be evaluated in view of correlation with symptoms of central sensitization.

Fibromyalgia comorbidity is a quite frequent cause of comorbidity in primary headache patients, characterizing more severe and invalidating forms. [17,18]. The present study confirms a high representation of FM syndrome in chronic tension type headache and chronic migraine and a rare presence in patients with TACs, migraine with aura and other primary headaches types [18].

In accord with sleep disorder as part of FM diagnosis [32,50], we found that headache patients with FM comorbidity presented with increased sleep disturbances and deprivation, which were both correlated with invalidity due to symptoms of diffuse pain. Present results may thus suggest that sleep problems other than raw sleep quantity are critical for FM comorbidity in primary headaches, while shortness of sleep alone prevails in allodynic not fibromyalgic headache patients. Nevertheless in FM patients, the lack of a linear correlation between sleep disturbances and somatic hyperalgesia may be due to the complex physiopathology of this syndrome, presently under careful revision for a possible involvement of peripheral afferents dysfunction [51].

## Conclusions

Summarizing, our study confirms that duration of sleep is associated to symptoms of central sensitization as allodynia and pericranial tenderness in primary headache patients. The association among allodynia, pericranial tenderness and short sleep characterizes chronic migraine more than any other primary headache form. Patients presenting with FM comorbidity suffer from a global impairment of sleep. Self reported duration of sleep seems a useful index to be correlated with allodynia, pericranial tenderness and chronic headache as a therapeutic target to be assessed in future prospective studies aiming to prevent central sensitization symptoms development.

## Abbreviations

SLPQRAW: Sleep quantity -raw; SLP6: Sleep index 1; TTS: Total tenderness score; SAS: Self rating anxiety scale; SDS: Self rating depression scale.

## Competing interests

The authors declare that they have no competing interests.

## Authors’ contributions

MD: study design, manuscript preparation and editing, statistical analysis, clinical assessment. MDE Psychological assessment, database management. EV: clinical assessment. VS: clinical assessment, manuscript preparation. SI: statistical analysis, manuscript editing. PL clinical assessment, manuscript editing. All authors read and approved the final manuscript.

## Authors’ information

MD: Associate Professor of Neurology, Head of Neurophysiopathology of Pain SMBNOS Department Bari University.

MDE: Psychologist, PhD, expert in pain assessment and management.

EV: Neurologist.

SI: Psychologist at Salento University, Aggregate Professor of General Psychologist.

PL. Full Professor of Neurology, Head of Neurological Clinic, SMBNOS Department.

## Supplementary Material

Additional file 1 Results of statistical analysis applied to all Medical Outcome Study Scale items: SLPD4: sleep disturbance, SLPSNR1: snoring, SLPSOB1: sleep short of breath or headache, SLPA2: sleep adequacy, SLPS3: sleep somnolence, SLP6: sleep problems index I, SLP9: sleep problems index II, SLPQRAW (sleep quantity -raw). Click here for file
