# Supplementary material for: Sleep features and central sensitization symptoms in primary headache patients
Source: J Headache Pain. 2014 Sep 26;15(1):64. doi: 10.1186/1129-2377-15-64 (PMC4189547; doi:10.1186/1129-2377-15-64)
Supplement: Additional file 1 — Results of statistical analysis applied to all Medical Outcome Study Scale items: SLPD4: sleep disturbance, SLPSNR1: snoring, SLPSOB1: sleep short of breath or headache, SLPA2: sleep adequacy, SLPS3: sleep somnolence, SLP6: sleep problems index I, SLP9: sleep problems index II, SLPQRAW (sleep quantity -raw). [file 1129-2377-15-64-S1.docx]

| Headache type  (IHS code) | | SLPD4  M | SD | SLPSNR1  M | SD | SLPSOB  M | SD | SLPA2  M | SD | SLPS3  M | SD | SLP6  M | SD | SLP9  M | SD | SLPQ  RAW M | SD |
| --- | --- | --- | --- | --- | --- | --- | --- | --- | --- | --- | --- | --- | --- | --- | --- | --- | --- |
|  | 1.1 | 43.45 | 22.13 | 49.22 | 39.37 | 47.18 | 32.55 | 50.27 | 32.24 | 50.65 | 29.11 | 50.39 | 24.92 | 50.67 | 28.50 | 6.67 | 1.510 |
|  | 1.2 | 37.13 | 260.07 | 49.63 | 44.85 | 44.44 | 38.16 | 55.56 | 34.57 | 46.42 | 33.92 | 44.81 | 29.55 | 48.64 | 30.76 | 70.07 | 1.174 |
|  | 1.1 + 2.1 | 47.78 | 24.38 | 53.52 | 39.25 | 46.48 | 31.85 | 47.62 | 31.64 | 54.74 | 28.99 | 53.19 | 25.33 | 54.27 | 29.91 | 6.83 | 1.331 |
|  | other | 48.37 | 17.85 | 62.76 | 39.54 | 52.41 | 36.81 | 41.38 | 320.04 | 56.32 | 29.25 | 57.47 | 23.24 | 60.92 | 26.53 | 6.45 | 1.270 |
|  | 1.1 +1.2 | 440.08 | 21.47 | 55.51 | 39.43 | 41.22 | 330.02 | 49.39 | 28.46 | 53.74 | 25.69 | 51.56 | 22.43 | 530.05 | 25.12 | 70.02 | 1.548 |
|  | 3.1 | 47.43 | 24.28 | 54.55 | 33.91 | 51.82 | 38.38 | 600.00 | 30.71 | 590.09 | 29.48 | 54.39 | 26.65 | 560.06 | 28.33 | 5.95 | 20.058 |
|  | 3.2. 3.4 | 40.34 | 22.42 | 34.55 | 36.98 | 41.82 | 37.37 | 56.36 | 310.07 | 39.39 | 25.90 | 42.12 | 27.82 | 39.39 | 29.58 | 6.73 | 1.272 |
|  | 1.3 | 47.90 | 22.40 | 51.69 | 40.56 | 53.39 | 34.63 | 48.73 | 30.87 | 50.91 | 26.46 | 51.69 | 21.35 | 520.08 | 25.24 | **60.08**  *******  **°**  **§§**  **^^^** | **1.634** |
|  | 2.2 | 45.76 | 22.81 | 44.17 | 41.10 | 49.17 | 290.06 | 47.64 | 30.83 | 52.45 | 29.19 | 530.06 | 22.75 | 53.43 | 26.68 | 6.96 | 1.578 |
|  | 2.3 | 45.74 | 23.55 | 540.05 | 38.85 | 500.00 | 32.48 | 45.95 | 31.71 | 56.13 | 28.84 | 530.08 | 20.91 | 52.56 | 25.76 | 6.28 | 1.469 |

Table 1 Mean (M) + Standard Deviation (SD) of MOS items in primary headache patients. Results of Bonferroni test are shown: **1.3 vs : ***** **1.1 p<0.001, ° 1.1 + 2.2 p<0.05 , §§ 1.1 + 1.2 p<0.01, ^^^ 2.2 p<0.001.**

- 1. : migraine without aura, 1.2: migraine with aura, 3.1 cluster headache,3.2 paroxysmal migraine,3.4 emicrania continua,1.3 chronic migraine,2.2 episodic tension type headache, 2.3 chronic tension type headache, OTHER: other primary headaches

SLPD4 : sleep disturbance , SLPSNR1 : snoring , SLPSOB1 : sleep short of breath or headache , SLPA2 : sleep adequacy , SLPS3 : sleep somnolence , SLP6 : sleep problems index I , SLP9 : sleep problems index II , SLPQRAW (sleep quantity -raw).

|  | | | DURATION | FREQUENCY | MIDAS | SAS | SDS | TTS | ALLODYNIA |
| --- | --- | --- | --- | --- | --- | --- | --- | --- | --- |
|  | Spearman  Rho | SLPD4 | 0.066^*^ | 0.069^*^ | **0.089^**^** | **0.143^**^** | **0.138^**^** | 0.048 | 0.023 |
|  |  | SLPSNR1 | -0.015 | 0.047 | -0.022 | 0.041 | **0.080^**^** | -0.018 | -0.028 |
|  |  | SLPSOB1 | -0.006 | **0.098^**^** | 0.070^*^ | **0.152^**^** | **0.132^**^** | 0.076^*^ | 0.071^*^ |
|  |  | SLPA2 | -0.035 | -0.058^*^ | -0.042 | **-.114^**^** | **-0.083^**^** | -0.025 | -0.049 |
|  |  | SLPS3 | -0.016 | 0.041 | 0.016 | -0.007 | -0.017 | 0.004 | -0.020 |
|  |  | SLP6 | 0.008 | 0.047 | 0.028 | **0.081^**^** | 0.040 | 0.057 | 0.010 |
|  |  | SLP9 | 0.004 | 0.036 | 0.004 | 0.030 | -0.006 | 0.057 | 0.003 |
|  |  | SLPQRAW | **-0.172^**^** | **-0.144^**^** | **-0.141^**^** | **-.0199^**^** | **-0.226^**^** | **-.0114^**^** | **-0.082^**^** |

Table 2 Spearman correlation test among MOS items and main clinical features in our cohort of 1670 primary headache patients.

The Rho values with a level of significance <0.01 (**) , were indicated in bold. (* p<0.05)

SAS: Self rating Anxiety Scale, SDS: Self rating Depression Scale, SLPD4 : sleep disturbance , SLPSNR1 : snoring , SLPSOB1 : sleep short of breath or headache , SLPA2 : sleep adequacy , SLPS3 : sleep somnolence , SLP6 : sleep problems index I , SLP9 : sleep problems index II , SLPQRAW sleep quantity, raw.

|  | | Type of headache. according to IHS classification code | | | | | | | | | | Total |
| --- | --- | --- | --- | --- | --- | --- | --- | --- | --- | --- | --- | --- |
|  |  | 1.1 | 1.2 | 1.2 + 2.2 | OTHER | 1.1 + 1.2 | 3.1 | 3.2. 3.4 | 1.3 | 2.2 | 2.3 |  |
| Number of cases | NO FM | 701 | 36 | 92 | 40 | 55 | 28 | 16 | 226 | 93 | 90 | 1377 |
|  | FM | 100 | 2 | 10 | 1 | 12 | 0 | 1 | 106 | 18 | 43 | 293 |
| Percent | | 12.4% | 5.2% | 9% | 2.4% | 17.2% | 0% | 5.8% | 31.92% | 16.21% | 32.33% | 17.54% |

Table 6 Frequency of Fibromyalgia (FM) diagnosis in primary headaches patients

1.1 : migraine without aura, 1.2migraine with aura, 3.1 cluster headache, 3.2 paroxysmal migraine, 3.4 emicrania continua, 1.3 chronic migraine, 2.2 episodic , tension type headache, 2.3 chronic tension type headache, OTHER: other primary headaches

|  | | | | | |
| --- | --- | --- | --- | --- | --- |
| MOS ITEMS | | MEAN | Standard error | 95% confidence interval | |
|  |  |  |  |  |  |
| SLPD4  SLPQRAW | NO FM | 44.39 | .71 | 42.99 | 45.79 |
|  | FM | 48.62 | 1.64 | 45.39 | 51.84 |
|  | NO FM | 6.73 | 0.09 | 6.55 | 6.92 |
|  | FM | 5.98 | .21 | 5.55 | 6.41 |

Table 7 Mean values of SLPD4 (sleep disturbances ) and SLPQRAW (sleep quantity –hours of sleep) in primary headache patients with and without fibromyalgia (FM) comorbidity. The Least Significance Difference (LSD) was 0.019 for SLPD4 and 0.002 for SLPQRAW.

|  | | SLPD4 | SLPSNR1 | SLPSOB1 | SLPA2 | SLPS3 | SLP6 | SLP9 | SLPQRAW |
| --- | --- | --- | --- | --- | --- | --- | --- | --- | --- |
| Spearman rho | Tender point count | 0.017 | 0.118 | 0.210^*^ | 0.089 | -0.053 | 0.087 | 0.035 | -0.249^*^ |
|  | FIQ | 0.110^*^ | **0.208^**^** | **0.156^**^** | -0.070 | **0.167^**^** | **0.284^**^** | **0.263^**^** | **-0.302^**^** |

Table 8 Spearman correlation test among MOS items and main clinical features of FM patients (n° 293) .

The Rho values with a level of significance <0.01 (**) , were indicated in bold. (* p<0.05).

FIQ: Fibromyalgia Impact Questionnaire, SLPD4 : sleep disturbance , SLPSNR1 : snoring , SLPSOB1 : sleep short of breath or headache , SLPA2 : sleep adequacy

SLPS3 : sleep somnolence , SLP6 : sleep problems index I , SLP9 : sleep problems index II , SLPQRAW (sleep quantity -raw)

|  | MOS items | |
| --- | --- | --- |
|  | NO FM | FM |
| SLPQRAW | 2.82 | 2.54 |
| (Constant) | -100.08 | -8.29 |

|  | Clinical features | |
| --- | --- | --- |
|  | NO FM | FM |
| SAS | 0.57 | 0.64 |
| TTS | 00.06 | 0.26 |
| MAF | -00.04 | -00.03 |
| (Constant) | -11.50 | -16.30 |

Table 9 Discriminating MOS items (top) and SLPQRAW plus other clinical features (bottom) between primary headache presenting and not presenting with fibromyalgia (FM) comorbidity.

Fisher’s linear discriminant functions

SLPQRAW sleep quantity, raw., SAS: : Self rating Anxiety Scale, SDS: MAF: Multidimensional Assessment of Fatigue, TTS: Total tenderness score.
